# Compressive Stress Induces Dephosphorylation of the Myosin Regulatory Light Chain via RhoA Phosphorylation by the Adenylyl Cyclase/Protein Kinase A Signaling Pathway

**DOI:** 10.1371/journal.pone.0117937

**Published:** 2015-03-03

**Authors:** Kenji Takemoto, Seiichiro Ishihara, Takeomi Mizutani, Kazushige Kawabata, Hisashi Haga

**Affiliations:** 1 Transdisciplinary Life Science Course, Faculty of Advanced Life Science, Hokkaido University, Sapporo, Japan; 2 Research Center for Cooperative Projects, Hokkaido University Graduate School of Medicine, Sapporo, Japan; Beatson Institute for Cancer Research Glasgow, UNITED KINGDOM

## Abstract

Mechanical stress that arises due to deformation of the extracellular matrix (ECM) either stretches or compresses cells. The cellular response to stretching has been actively studied. For example, stretching induces phosphorylation of the myosin regulatory light chain (MRLC) via the RhoA/RhoA-associated protein kinase (ROCK) pathway, resulting in increased cellular tension. In contrast, the effects of compressive stress on cellular functions are not fully resolved. The mechanisms for sensing and differentially responding to stretching and compressive stress are not known. To address these questions, we investigated whether phosphorylation levels of MRLC were affected by compressive stress. Contrary to the response in stretching cells, MRLC was dephosphorylated 5 min after cells were subjected to compressive stress. Compressive loading induced activation of myosin phosphatase mediated via the dephosphorylation of myosin phosphatase targeting subunit 1 (Thr853). Because myosin phosphatase targeting subunit 1 (Thr853) is phosphorylated only by ROCK, compressive loading may have induced inactivation of ROCK. However, GTP-bound RhoA (active form) increased in response to compressive stress. The compression-induced activation of RhoA and inactivation of its effector ROCK are contradictory. This inconsistency was due to phosphorylation of RhoA (Ser188) that reduced affinity of RhoA to ROCK. Treatment with the inhibitor of protein kinase A that phosphorylates RhoA (Ser188) induced suppression of compression-stimulated MRLC dephosphorylation. Incidentally, stretching induced phosphorylation of MRLC, but did not affect phosphorylation levels of RhoA (Ser188). Together, our results suggest that RhoA phosphorylation is an important process for MRLC dephosphorylation by compressive loading, and for distinguishing between stretching and compressing cells.

## Introduction

Tissues and cells in the human body are continually subjected to mechanical stress, such as compression, shear flow, and pressure. In order to maintain homeostasis in the body, it is necessary for cells to respond to mechanical stress. If cells cannot adequately respond to stress, the body will deteriorate into a pathological state. For example, vascular smooth muscle cells dilate and constrict in response to variations in blood pressure. High blood pressure causes an increase in blood flow and shear stress on vascular endothelial cells resulting in vascular damage. On the other hand, low blood pressure reduces blood circulation making it more difficult to provide nutrients and oxygen throughout the body. Therefore, in order to maintain constant blood flow, smooth muscle cells contract or dilate in response to high or low blood pressure [1], [2]. Dysfunctional response to blood pressure induces several vascular diseases, such as hypertension [3], chronic heart failure [4] and vasospastic angina [5]. Thus, elucidating the cellular response to different mechanical stress in the human body is important for understanding various physiological events, including disease development.

Mechanical stress that arises due to deformation of extracellular matrix (ECM) is divided broadly into two categories: stretch and compression. The cellular response to stretching cells has been widely studied. The intracellular factors sensing cell stretching are membrane-associated proteins (integrins [6] and cation channels [7]) and cytoskeletons (actin filaments [8] and microtubles [9]). Signaling pathways are activated by these factors, and cells respond through the production of extracellular matrix [10], cell differentiation [11], and increases in cellular elasticity [12]. On the other hand, cellular response to compressive loading are not well understood. It is also unclear which proteins can sense differences between stretching and compressing cells.

Stretching cells also affects cellular tension force. Actin fibers, which are known as sensors of mechanical stress, exert traction force by interacting with myosin II. The tensional force is regulated by phosphorylation of myosin regulatory light chain (MRLC) at Thr18 and Ser19, which stimulates the ATPase activity of myosin II [13], [14]. The phosphorylation levels of MRLC are regulated by the balance between activities of some kinases and myosin light chain phosphatase (MLCP). MLCP consists of three subunits, a catalytic subunit of the type 1 protein serine/threonine phosphatase family (PP1δ), a myosin phosphatase targeting subunit (MYPT1), and a 20-kDa small subunit (M20) [15], [16]. MYPT1 is phosphorylated by ROCK, an effector of RhoA, and thereby inactivates MLCP [17]. After cells were forced to stretch, MRLC was phosphorylated via activation of RhoA and ROCK, and cells exerted stronger traction forces [18]. However, it is unknown whether MRLC is phosphorylated or dephosphorylated in response to compressive loading.

In this study, we investigated how cells sense the differences between stretching and compression by examining the response of MRLC to compressive loading. We determined whether the levels of phosphorylated MRLC were changed in response to compressive loading using a previously developed device that compresses cells isotropically. As a result, MRLC was dephosphorylated in response to compressive loading in contrast to the response of MRLC when stretching cells. The MRLC dephosphorylation response was induced by MLCP activation, MYPT1 dephosphorylation, and ROCK inactivation. Interestingly, compressive loading induced RhoA activation that was similar to the RhoA response to stretching cells. It was reported that the phosphorylation of RhoA (Ser188) reduces the affinity of RhoA to ROCK [19]. Western blotting showed that RhoA was phosphorylated in response to compressive loading. In contrast, the phosphorylation levels of RhoA did not oscillate by stretching cells. The phosphorylation of RhoA after compressing cells was induced by the adenylyl cyclase/protein kinase A (PKA) signaling pathway. These results suggest that the mechanisms that regulate cellular tension in response to variations in mechanical stress are controlled by the presence or absence of RhoA phosphorylation by PKA, but not activation or inactivation of RhoA.

## Materials and Methods

### Cell culture

Murine C2C12 skeletal myoblast cells were obtained from the RIKEN Cell Bank (Tsukuba, Japan). Cells were cultured in Dulbecco’s modified Eagle’s medium (DMEM; Sigma-Aldrich, MO, USA) supplemented with 10% fetal bovine serum (BIST-TEC; Equitech Bio Inc., TX, USA) and 1% antibiotic solution (Sigma-Aldrich) at 37°C in a humidified atmosphere of 5% CO_2_.

### Reagents

Protein phosphatase 1 and 2A inhibitor (Calyculin A), protein kinase A inhibitor (H-89), and adenylyl cyclase inhibitor (SQ22,536) were purchased from Sigma-Aldrich. The following primary antibodies were used for western blotting: myosin light chain 2 antibody (#3672; Cell Signaling Technology, MA, USA), phospho-myosin light chain 2 (Ser19) antibody (#3671; Cell Signaling Technology), phospho-myosin light chain 2 (Thr18/Ser19) antibody (#3674; Cell Signaling Technology), myosin light chain 2 antibody (#3672; Cell Signaling Technology), GAPDH antibody (#AM4300; Ambion, TX, USA), MYPT1 antibody (#2634; Cell Signaling Technology), phospho-MYPT1 (Thr696) antibody (#5163; Cell Signaling Technology), phospho-MYPT1 (Thr853) antibody (#4563; Cell Signaling Technology), RhoA antibody (#ARH03; Cytoskeleton, CO, USA), and phospho-RhoA (Ser188) antibody (#AB41435; Abcam, Cambridge, UK). MRLC stained by phospho-myosin light chain 2 (Ser19) antibody is denoted as 1P-MRLC, and MRLC stained by phospho-myosin light chain 2 (Thr18/Ser19) antibody is denoted as 2P-MRLC.

### Application of mechanical strain

**Fig 1:**
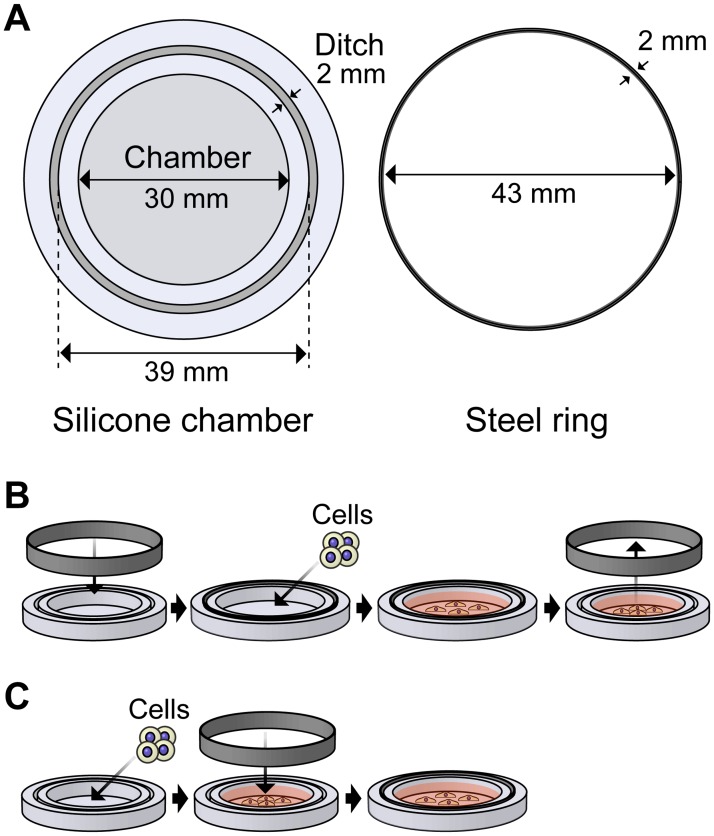
Methods for application of mechanical strain. (A) An overview of the isotropic deformation device consisting of a silicone chamber and a steel ring. When the steel ring was inserted into the silicone ditch, the silicone chamber was 39 mm in diameter. (B) Method for compressing cells. A silicone chamber is pre-extended by inserting a steel ring into a ditch. Cells are seeded on the pre-extended silicone chamber, and compressed by pulling out a steel ring from a ditch. (C) Method for stretching cells. Cells are cultured on a silicone chamber without inserting a steel ring. By inserting a steel ring into a ditch, cells are stretched.

Cells were stretched or compressed isotropically using previously described methods [20]. The silicone chamber and steel ring were prepared, as shown Fig. 1A. The chamber is made of a transparent silicone rubber (SH9555; Toray Dow Corning Silicone, Tokyo, Japan). For cell compression, the silicone chamber was pre-stretched by inserting the steel ring into the ditch of silicone. Myoblast cells were removed from the culture dishes using trypsin-EDTA, transferred to the chamber, and cultured on pre-stretched silicone coated with 50 μg/mL fibronectin. When the ring was pulled out of the ditch, the cells were compressed via the compression of the silicone chamber (Fig. 1B). We observed that cell area was decreased 12% immediately after cell compression (S2C Fig.). In order to perform cell stretching, cells were cultured on the silicone chamber which was not pre-stretched, and then stretched by inserting the steel ring into the ditch of silicone (Fig. 1C). The medium was replaced with DMEM containing 1% FBS and 1% antibiotic solution for 3 hours before applying mechanical strain. The cells were treated with the following inhibitors for 90 minutes before mechanical loading: 2 nM calyculin A (Sigma-Aldrich), 30 μM H-89 (Sigma-Aldrich), and 100 μM SQ22,536 (Sigma-Aldrich).

### Western blotting

Cells were fixed in ice-cold trichloroacetic acid for 3 minutes and washed 3 times with PBS. Cell lysates were prepared in Laemmli sample buffer (0.25 M Tris-HCl, 5% dithiothreitol, 2.3% sodium dodecyl sulfate, 10% glycerol, 0.01% bromophenol blue, pH 8.8). The proteins were then separated by SDS-PAGE using 7.5% polyacrylamide gels (for MYPT1) and 12.5% (for other proteins). After blocking (5% skimmed milk in TBS-Tween 20 solution, 10 mM Tris-HCl containing 150 mM NaCl and 0.05% Tween 20, pH 7.5), the blots were incubated at 4°C overnight with the appropriate primary antibody. After incubation with a secondary antibody (HRP-conjugated anti-mouse or anti-rabbit IgG), the blots were developed using Can Get Signal Immunoreaction Enhancer Solution (Toyobo, Osaka, Japan). Protein levels of GAPDH were used as an internal standard. Western blots were quantified by Image J software (National Institutes of Health, Bethesda, MA).

### Observation of actin filaments

To observe the dynamics of F-actin in cells under compressive loading, we performed live cell imaging. Semi-confluent C2C12 cells on a culture dish were transfected with a previously constructed plasmid encoding azami green-tagged β-actin [21] and cultured for 1 day. The cells were removed from the culture plate using trypsin-EDTA, transferred onto the silicone chamber, and observed with a Nikon C1 confocal imaging system (Nikon Instech., Tokyo, Japan).

To assess the thickness of F-actin in cells under compressive strain, we performed immunofluorescent staining. Cells were fixed with 4% paraformaldehyde in PBS for 10 minutes, permeabilized with 0.5% Triton X-100 in PBS for 10 minutes. The samples were then blocked with 0.5% BSA in PBS for 1 hour at room temperature, and incubated with MFP 488-phalloidin for 1 hour at room temperature. We observed the samples after treatment with a fade-resistant solution (2.5% DABCO, 90% glycerol, 6% PBS; pH 8.0). The resulting images were captured with a Nikon C1 confocal imaging system using the ×60 objective (Nikon Instech.).

### Quantitative analysis of actin filament thickness

The thickness of F-actin was quantitatively analyzed with a stress fiber thickness index (SFTI) [22]. In brief, immunofluorescent images of actin filaments were subjected to a minimum filter, which replaced each pixel in the image with the smallest pixel value in that pixel’s neighborhood. Fluorescence intensities on F-actin were gradually decreased by repeating minimum filtering. Actin filament intensity against the number of minimum filtering was fitted using the following equation:

$$I=A+B\cdot \exp\left(-\frac{N}{\tau }\right)$$

where *I* is fluorescence intensity on a F-actin image, *N* is the number of the minimum filtering, *τ* is the decay constant, and *A* and *B* are constants. The fluorescence intensity of thicker fibers decayed slower with filtering; thus, the decay constant was associated with actin filament thickness. The decay constant *τ* was used as a SFTI. Images of phalloidin-stained actin filaments in 60 individual cells were randomly selected from 3 independent experiments and analyzed.

### RNA interference (RNAi)

RNAi was used to knockdown the expression of the MYPT1 gene. The target sequence of the siRNA used in this study was 5′-CUGUGGAUAUCUCGAUAUUGC-3′ (sense sequence). As a negative control, the following non-target sequence of the siRNA was randomly selected and used: 5′-ACUACAUGUCACAUCACGGUU-3′ (sense sequence). Cells were transfected with siRNA duplexes using Lipofectamine RNAiMAX (Invitrogen, CA, USA) and were used for the designated assay after incubation for 2 days. The silencing efficiency was confirmed by western blotting.

### RhoA activation assay

The Rho activity assay was performed and quantified using the RhoA Activation Assay Biochem Kit (Cytoskeleton) based on the Rhotekin pull-down assay. In brief, cells were washed with PBS then extracted using cell lysis buffer (50 mM Tris, pH 7.5, 10 mM MgCl_2_, 0.5 M NaCl, 2% Igepal). Samples were centrifuged for 5 minutes at 10,000 × *g* and then the supernatant was incubated with Rhotekin-RBD beads for 1.5 hours at 4°C. After washing the beads with buffer (25 mM Tris, pH 7.5, 30 mM MgCl_2_, 40 mM NaCl), proteins were removed from the beads with Laemmli buffer, then subjected to western blotting.

### Statistical analysis

Statistical analysis of the western blot data was performed using the Student’s *t*-test. Data is presented as mean ± standard error of the mean (SEM), and consists of 3 independent experiments.

## Results

### MRLC dephosphorylation in response to cell compression

**Fig 2:**
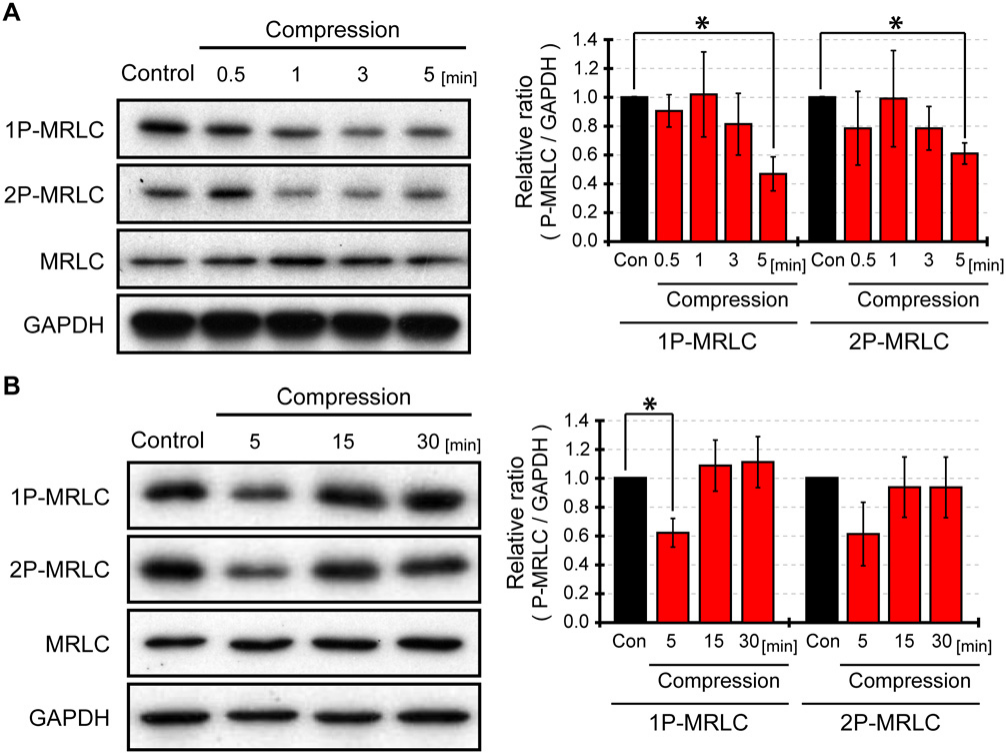
Phosphorylation of myosin regulatory light chain in response to compression. The phosphorylation levels of myosin regulatory light chain (MRLC) in C2C12 cells stimulated by 12% compression was detected by western blotting. Mono-phosphorylated MRLC (Ser19) is expressed as 1P-MRLC, and di-phosphorylated (Thr18/Ser19) is 2P-MRLC. Cells without compression were used as a control, and the compressed cells were analyzed at 0.5, 1, 3, and 5 minutes (A) and 5, 15, and 30 minutes (B) after compression. The levels of 1P-MRLC, 2P-MRLC, MRLC and GAPDH were quantified with Image J software. Quantification of western blots represents an average of 3 independent experiments, mean ± SEM. *, *p < 0*.*05*.

We examined whether the phosphorylation levels of myosin regulatory light chain (MRLC) in C2C12 cells changed after 12% cell compression. In order to investigate time-dependent changes in MRLC phosphorylation, we analyzed mono-phosphorylated MRLC at Ser19 (1P-MRLC) and di-phosphorylated MRLC at Thr18 and Ser19 (2P-MRLC) by western blot at 0.5, 1, 3, and 5 minutes after compression (Fig. 2A). The levels of phosphorylated MRLC 5 minutes after compressive loading decreased relative to the control without compressive stress. To determine whether MRLC underwent continued dephosphorylation for more than 5 minutes after compressive loading, we detected phosphorylated MRLC at 5, 15, and 30 minutes after cell compression (Fig. 2B). Similar to Fig. 2A, MRLC was dephosphorylated 5 minutes after cell compression. Fifteen minutes after compressive loading MRLC was more phosphorylated than at 5 minutes after cell compression. Furthermore, 15 minutes after cell compression the level of MRLC phosphorylation returned to the level reported before cell compression. Therefore, MRLC was significantly dephosphorylated in C2C12 cells 5 minutes after compression to 12%, but returned to the control levels by 15 minutes after compressive loading.

### 12% compression did not affect actin filaments in C2C12 cells

**Fig 3:**
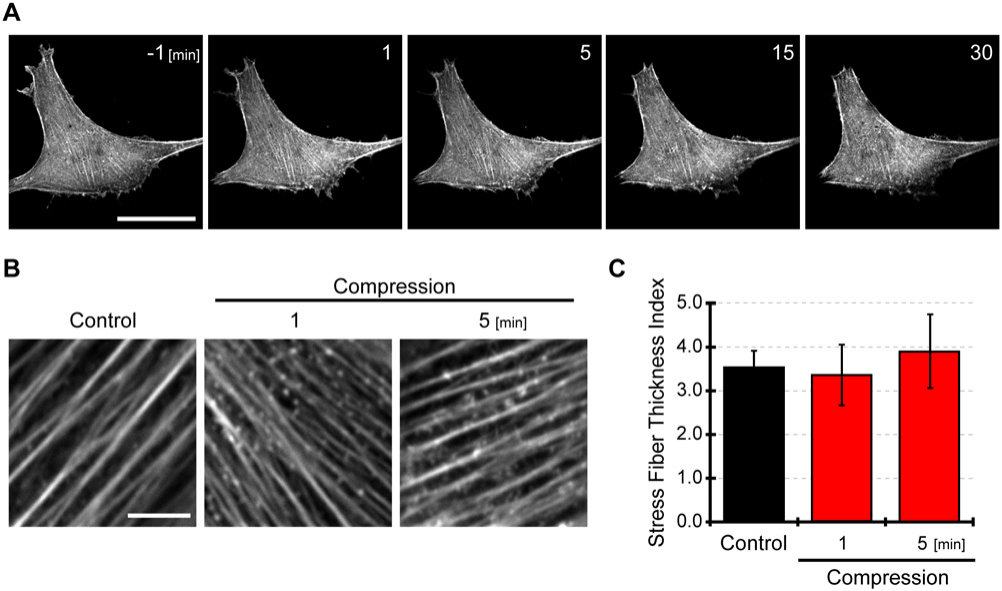
F-actin was unaffected by compressive stress. (A) Live imaging of cells expressing azami green-actin under compressive loading. Numbers at upper right in images are relative time (min) from the onset of compressive loading. Scale bar is 50 μm. (B) Immunofluorescent imaging of F-actin in cells before and after induction of compression. We observed F-actin stained with MFP 488-phalloidin in cells before and after compressive strain. Scale bar is 5 μm. (C) Stress fiber thickness index in cells under compressive stress. By analyzing immunofluorescent images of F-actin, we assessed the thickness of F-actin in the form of stress fiber thickness index. The thicker actin filaments have a larger value of stress fiber thickness index. Image J software was used to estimate the thickness of F-actin. The stress fiber thickness index was calculated from 3 independent experiments including 60 individual cells. The results were presented as mean ± SEM.

Next, we explored the mechanism of MRLC dephosphorylation in response to cell compression. A previous study reported that F-actin disruption with cytochalasin D induced MRLC dephosphorylation [23]; therefore, we observed the behavior of actin filaments in cells expressing azami green-acitn (Fig. 3A). Live cell imaging showed that F-actin was not disrupted after cell compression. Even without significant disruption of actin filaments, the thickness of actin filaments might change in response to compressive loading. The thickness of actin filaments was quantitatively assessed using immunofluorescent techniques (Fig. 3B). Actin filament thickness was estimated by the stress fiber thickness index (SFTI). SFTIs were not statistically different before or after compressive loading (Fig. 3C). Thus, these results suggested that 12% compressive stress did not affect F-actin dynamics.

### Compression induced MRLC dephosphorylation via activation of myosin light chain phosphatase

**Fig 4:**
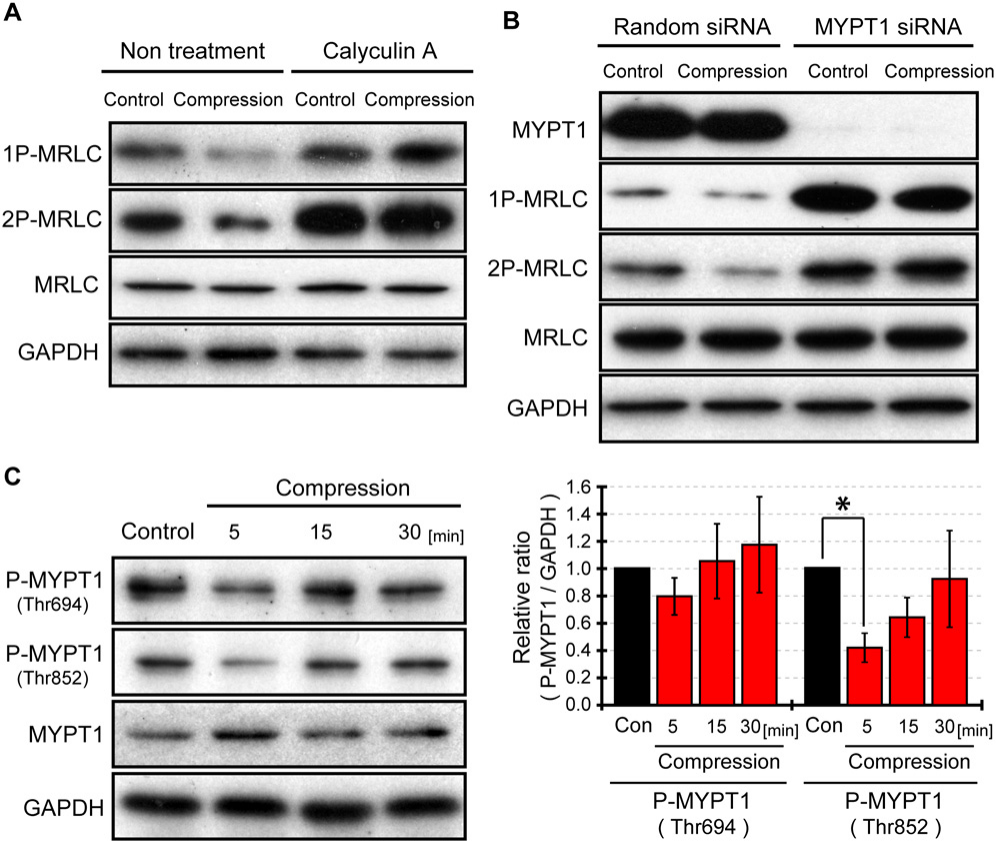
Compression induced inactivation of ROCK, and activated MLCP. (A) Influence of MLCP inhibition on phosphorylated MRLC under compressive stimulation. Cells were compressed 90 minutes after the treatment of calyculin A (2 nM). Uncompressed cells were used as a control, and compressed samples were taken from cells 5 minutes after compression. (B) Effect of siRNA-mediated knockdown of MYPT1 on phosphorylated MRLC under compressive loading. Cells were transfected with random or MYPT1 siRNA 24 hours before cell compression. 1P-MRLC, 2P-MRLC, and GAPDH were measured by western blotting. (C) Western blotting for phosphorylated MYPT1 under cell compression. MYPT1 (Thr696) and MYPT1 (Thr853) dephosphorylation 5 minutes after cell compression were detected by western blotting. The levels of phospho-MYPT1 and GAPDH were quantified with Image J software. Quantification of western blots represents an average of three independent experiments, mean ± SEM. *, *p < 0*.*05*.

Only one type of protein phosphatase (PP), called myosin light chain phophatase (MLCP), dephosphorylates MRLC. We examined the role of MLCP in MRLC dephosphorylation as a response to compressive loading. MLCP is composed of three subunits: a catalytic subunit (protein phosphatase 1cδ, PP1cδ), a myosin binding regulatory subunit (myosin phosphatase target subunit 1, MYPT1), and a subunit of unknown function (p20). First, the catalytic activity of MLCP was inhibited with calyculin A, a PP1 and PP2A inhibitor. Cells were treated with calyculin A (2 nM) 90 minutes before compressive loading, and then we examined whether MRLC was dephosphorylated in response to compressive stress (Fig. 4A). Accordingly, when compression was applied to cells under the treatment of calyculin A, MRLC was not dephosphorylated. Next, MYPT1, which is a subunit of MLCP was knocked down using RNAi (Fig. 4B). As a result, compression did not induce MRLC dephosphorylation in cells transfected with MYPT1 siRNA. Thus, these results suggested that MLCP activity was involved in MRLC dephosphorylation in response to compression.

In order to reveal how the MLCP is activated after cell compression, we studied how MYPT1 regulates MLCP activity. MYPT1 can bind to both MRLC and PP1cδ [24], [25]. MYPT1 facilitates the specific activity of PP1cδ in the dephosphorylation of phospho-MRLC by tethering the catalytic subunit to MRLC. Thr694 and/or Thr852 at MYPT1 can be phosphorylated. Phospho-MYPT1 is also a target of PP1cδ, and lowers substrate specificity of PP1cδ to MRLC [26], [27], [28]. Additionally, phosphorylation of MYPT1 (Thr852) interferes with the binding of MYPT1 to MRLC [29]. For these reasons, phosphorylation of MYPT1 inactivates MLCP. In contrast, blockage of MYPT1 phosphorylation induces MLCP activation. MYPT1 mutants (Thr694Ala and Thr852Ala) mimick the un-phosphorylated state, can enhance MLCP activity and decreases the levels of phosphorylated MRLC compared to wild type MYPT1 [30]. Because MLCP was activated after cell compression, we hypothesized that these MYPT1 sites are dephosphorylated in response to compressive loading. We examined the phosphorylation levels of MYPT1 (Fig. 4C). MYPT1 (Thr852) was significantly dephosphorylated after cell compression, whereas MYPT1 (Thr694) was not. These findings indicate that compression activated MLCP via MYPT1 (Thr852) dephosphorylation, which resulted in the dephosphorylation of MRLC. The Thr694 site of MYPT1 is phosphorylated by various kinases: ROCK, ZIPK, ILK, and PAK [26], [27], [28]. On the other hand, the Thr852 site of MYPT1 is phosphorylated only by ROCK [26]. Taken together, it may be suspected that ROCK was inactivated by compressive stress.

### RhoA activation and phosphorylation by compressive loading

**Fig 5:**
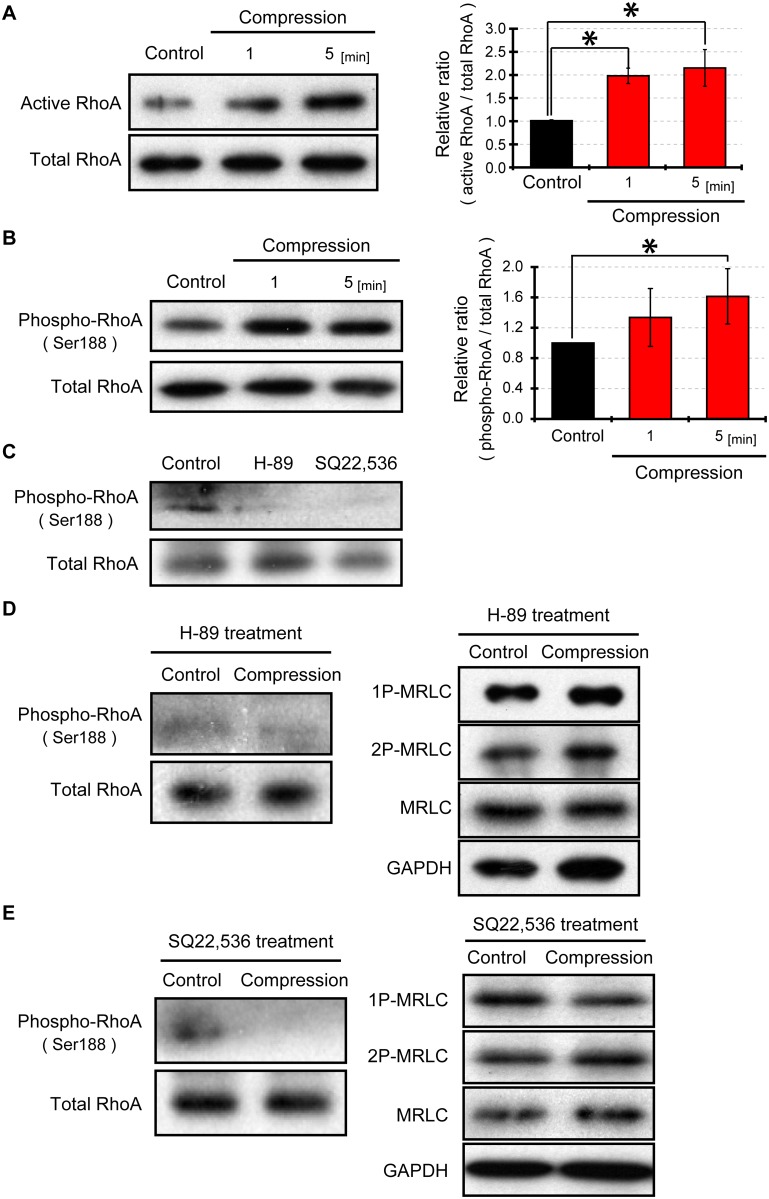
Compression induced RhoA activation and phosphorylation. (A) Detection of active RhoA under compressive loading. Active RhoA (RhoA-GTP) was precipitated with Rhotekin-RBD beads. Total RhoA was used as a loading control. (B) Western blotting for phosphorylated RhoA after compressing cells. RhoA (Ser188) phosphorylation was detected by western blotting, and total RhoA was used as a loading control. (C) Effects of protein kinase A (PKA) inhibitor H-89 or adenylyl cyclase inhibitor SQ22,536 on phosphorylated RhoA (Ser188). Cells on a plastic dish were treated with 30 μM H-89 or 100 μM SQ22,536 for 90 minutes. (D) Influence of PKA inhibition on compression-induced RhoA phoshorylation and MRLC dephosphorylation. Cells were compressed under the presence of 30 μM PKA inhibitor H-89. (E) Effect of inhibition of adenylyl cyclase on compression-stimulated RhoA phoshorylation and MRLC dephosphorylation. Cells were treated with 100 μM adenylyl cyclase inhibitor (SQ22,536) for 90 minutes before cell compression. The levels of active RhoA, phospho-RhoA, total RhoA, 1P-MRLC, 2P-MRLC, MRLC and GAPDH were detected by western blotting and quantified with Image J software. Quantification of western blots represents an average of 3 independent experiments, mean ± SEM. *, *p < 0*.*05*.

It was deduced that ROCK was inactivated in response to compressive loading. We examined whether compression induced inactivation of RhoA, which up-regulates ROCK. An immunoprecipitation technique with Rhotekin-RBD beads was used to detect the levels of RhoA-GTP (active RhoA) before and after cell compression. Interestingly, active RhoA was increased 1 minute after compressive stress (Fig. 5A). Here, there was a discrepancy between inactivated ROCK and activated RhoA after compression. Nusser et al. reported that when RhoA is phosphorylated at the Ser188 site, it cannot interact and activate ROCK [19]. We hypothesized that RhoA was phosphorylated in response to cell compression. Western blot results showed that RhoA (Ser188) was significantly phosphorylated 5 minutes after compressive loading (Fig. 5B).

### Adenylyl cyclase/PKA signaling regulated MRLC dephosphorylation response

It has been reported that RhoA (Ser188) is phosphorylated by protein kinase A (PKA) [31]. We investigated the contribution of RhoA phosphorylation by PKA to the MRLC dephosphorylation response. First, we examined the efffects of PKA inhibitor H-89 on the phosphorylation levels of RhoA. Cells on a plastic dish were treated with H-89 at a concentration of 30 μM for 90 minutes, and then, phosphorylation levels of RhoA was decreased by H-89 treatment (Fig. 5C). Next, we investigated the efects of the treatment of H-89 on compressive-induced RhoA phosphorylation and MRLC dephosphorylation. Cells on a silicone rubber were treated with H-89 (30 μM) 90 minutes before compressive loading. PKA inhibitor H-89 resulted in blockage of RhoA phosphorylation in response to cell compression (Fig. 5D). Additionally, there was no significant difference between the levels of phosphorylated MRLC in cells with and without compression when treated with PKA inhibitor (Fig. 5D). Therefore, these results suggest that RhoA phosphorylation induced by PKA leads to MRLC dephosphorylation in response to compressive loading.

The activity of PKA is dependent on intracellular cyclic adenosine monophosphate (cAMP) concentration [32]. cAMP is synthesized from adenosine triphosphate by adenylyl cyclase [33]. To examine whether inhibition of adenylyl cyclase suppresses MRLC dephosphorylation in response to compressive loading, we initially investigated the effects of adenylyl cyclase inhibitor (SQ22,536) on the phosphorylation levels of RhoA. When cells were treated with 100 μM SQ22,536 for 90 minutes, levels of phosphorylated RhoA were decreased (Fig. 5C). We subsequently investigated the effects of the inhibition of adenylyl cyclases on compressive-induced RhoA phosphorylation and MRLC dephosphorylation. Cells on a silicone rubber were treated with SQ22,536 (100 μM) 90 minutes before compressive loading. Under the treatment of SQ22,536, phosphorylation levels of RhoA were decreased rather than remaining unchanged by compressive loading (Fig. 5E). Moreover, inhibition of adenylyl cyclase blocked compression-stimulated MRLC dephosphorylation (Fig. 5E). These results suggest that the adenylyl cyclase/cAMP/PKA signaling pathway is involved in MRLC dephosphorylation response to compression.

### Stretching cells cannot induce phosphorylation of RhoA

The deformation stress opposite to compressive loading is cell stretching. The cellular response to stretching also contrasts the response to cell compression. For example, MRLC was phosphorylated via ROCK activation after fibroblast cells were subjected to stretching [18]. We confirmed that stretching induced MRLC phosphorylation in C2C12 cells as well (S1A Fig.). On the other hand, we found that the RhoA response to stretching was consistent with the response to compressive stress. We investigated the RhoA activity 5 minutes after C2C12 cells were stretched. Then RhoA was activated by stretching cells (S1B Fig.). Combined with the result that RhoA activation was induced by compressive loading, RhoA was activated in response to both stretching and compressing stress. In other words, it suggests that RhoA cannot distinguish stretching cells from compressive loading. We hypothesized that MRLC dephosphorylation response to cell compression as distinguished from stretching was caused by RhoA phosphorylation, but not due to change in RhoA activity. Western blotting for phosphorylated RhoA was used to examine whether phosphorylated RhoA changed in response to stretching cells. There was no significant difference in RhoA phosphorylation between stretched and unstretched cells (S1C Fig.). Therefore, RhoA phosphorylation may be the means by which MRLC can discriminate between stretched and compressed cells.

## Discussion

**Fig 6:**
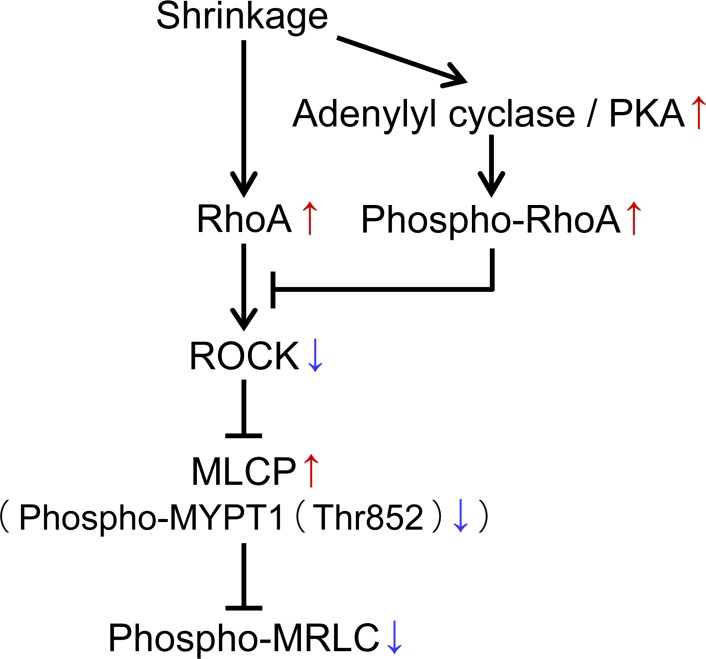
Proposed model for compression-induced dephosphorylation of myosin regulatory light chain (MRLC). In C2C12 cells subjected to compressive loading, RhoA and adenylyl cyclase activation were induced. Adenylyl cyclase activation may activate protein kinase A (PKA) via cAMP production. RhoA phosphorylation by PKA induces decreasing affinity of RhoA for ROCK and inactivates ROCK [19]. Myosin phosphatase target subunit 1 (MYPT1) at Thr852 which is kinase substrate of ROCK is dephosphorylated. Accordingly, myosin phosphatase (MLCP) is activated and dephosphorylates MRLC.

In this study, we obtained the following results. (1) Myosin regulatory light chain (MRLC) was dephosphorylated in C2C12 cells subjected to compressive loading. (2) MRLC dephosphorylation in response to cell compression was induced by MLCP activation via MYPT1 (Thr853) dephosphorylation. (3) Compression may induce ROCK inactivation despite RhoA activation. (4) The MRLC dephosphorylation response was due to RhoA phosphorylation through the adenylyl cyclase/protein kinase A signaling pathway. Fig. 6 is the signaling pathway summarizing the results suggested in the present work. Additionally, we hypothesize that RhoA phosphorylation is also an important process for distinguishing between stretching and compressing cells.

The finding that MRLC was dephosphorylated in response to compressive loading is thought to influence cell density. Keeping the cell density constant can help control tissue and organ size, and aberration of this system can trigger cancer development and other diseases. An increase in cell density due to excess cell division causes compressive stress on the cells themselves. In order to reduce the excessive cell density, cells must stop proliferating. Recently, it was reported that cells on a stiffer extracellular matrix (ECM) activate Yes-associated protein (YAP) and transcriptional coactivator with PDZ-binding motif (TAZ), which promotes cell proliferation and cell survival [34]. YAP/TAZ activation in response to ECM stiffness was mediated by MRLC phosphorylation. Thus, the MRLC dephosphorylation response to compression may inhibit cell proliferation signaling via YAP/TAZ. This suggestion is supported by the report that DNA synthesis scaled directly with projected cell area: cells on a small adhesive island coated with fibronectin showed low ability of cell proliferation and survival [35]. Additionally, George T. Eisenhoffer reported that increased density in MDCK cells was de-escalated through apical extrusion of cells and returned to the constant cell area [36]. Suspension of cell division through YAP/TAZ inactivation and apical extrusion of cells by compressive loading may regulate cell density *in vivo*.

We considered the effect of compressive loading on cell area. Cells can control their own size, and regulate their cell area to keep it constant. For example, immediately following cell division cells are very small, and then gradually grow to the size of their parent cell [37]. We examined how cell size changes in response to compressive loading when cells are forced to decrease in size. As a result, cells gradually grew to a large size after compressive stress and returned to their original size 20 minutes after cell compression (S2A Fig.). Initially, we thought that increasing cell size after compressive loading was due to MRLC dephosphorylation. However, cell area is regulated by cellular tension, which is dependent on the phosphorylation of MRLC, and time-dependent change of phosphorylated MRLC in response to compressive loading is correlated with the change in traction force during cell spreading [38]. Therefore, we investigated the cell area of cells that were compressed under the inhibition of adenylyl cyclase, which is important in the MRLC dephosphorylation response to compression. However, cells under the treatment of adenylyl cyclase inhibitor also return to their original size after compressive loading along with the non-treated cells (S2B,C Fig.). Thus, these results suggested that MRLC dephosphorylation by cell compression may not be necessary to regulate cell area.

Cell spreading is promoted by protrusion of membrane ruffles. Rho GTPases is implicated in the formation of actin filamients in membrane protrusion. A recent study showed that Rac1, which is one of the RhoGTPases, was essential for cell migration, but not for cell spreading [39]. On the other hand, RhoA is involved not only in contractile force generation by ROCK, but also membrane protrusion by mDia1. Using a FRET technique, it was reported that RhoA was activated 40 seconds before Rac1 activation in lamellipodia formation [40]. Therefore, the RhoA/mDia1 signaling pathway may be related to reconstruction of cell area after compressive loading.

Based on Fig. 3, we concluded that compressive loading did not induce collapse or thickness change of actin filaments. However, actin particles with approximately 0.5 μm diameter were observed after cell compression (Fig. 3B). There are three conceivable causes why the actin particles were generated. (i) Cortical thinner actin filaments may be disrupted. Hayakawa et al. reported that actin-severing protein cofilin selectively bind to unstrained actin fibers [41]. Since compressive loading decreased tension of actin fibers, enabling binding of cofilin to the the actin fibers and sebsequently severing them. Although disruption of actin fibers was not observed with live-cell imaging, observable actin fibers using this technique are thick bundles of actin filaments called stress fibers. Thus, thinner cortical actin filaments may be disrupted by cofilin, so that actin particles increased after cell compression. (ii) The actin particles are possibly the nucleus of actin polymerization. Compressive loading induced RhoA activation and can activate mDia1. mDia1 can stabilize actin dimers and trimers that are intermediates of actin polymerization [42]. Profilin interacts with mDia1 and recruits the actin monomers to the actin nucleus, therefore, actin filaments are elongated [43]. RhoA activation by compressive stress might induce activation of mDia1, resulting in nucleation of actin polymerization. (iii) The particles may be endosomes and exosomes. Endocytosis and exocytosis regulate not only composition and surface area, but also tension of cellular membranes. Endocytosis increases membrane tension by removing excess membrane components, whereas exocytosis relaxes the cell membrane. In this study, compressive loading decreased cellular membrane tension. In order to maintain the plasma membrane tension, endocytosis may be stimulated after cell compression. Additionally, clathrin-mediated endocytosis is associated with the actin cytoskeleton. Actin dynamics is required for membrane invagination and clathrin-mediated plaque uptake [44]. Actin and some of its interacting proteins (Arp2/3, cortactin, Hip1R etc.) are recruited to clathrin-containing structures upon scission from the plasma membrane [45], [46]. Hence, it would not be surprising if endocytosis was activated by change in the tension of the actin cytoskeleton after cell compression.

In this study, RhoA was activated after induction of compressive stimuli. Furthermore, a previous study revealed that stretching cells also induced RhoA activation [18]. In summary, cells cannot sense differences between compressing and stretching cells in terms of RhoA activity. Some guanine nucleotide exchange factors (GEF), which are activators for RhoA, are known to respond to mechanical stress. For example, LARG and GEF-H1 were activated in response to generate tensile forces on fibronectin-coated magnet beads attached to the dorsal surface of cells [47]. Vav-2 which can exchange GDP for GTP on several Rho-GTPases including RhoA, Rac1, RhoG, and Cdc42 responded to fluid shear flow and ECM stiffness [48], [49]. Alternatively, p190RhoGEF [50] and PDZRhoGEF [51], also known as RhoGEFs, were activated by mechanical stress. These RhoGEFs may sense compressive stress and activate RhoA.

This study reported that adenylyl cyclase was involved with MRLC dephosphorylation in response to compressing cells. Ten types of adenylyl cyclases have been found, of which eight types are expressed in mouse. Adenylyl cyclase activity is altered by some agents. All adenylyl cyclase isozymes are stimulated by forskolin [52], seven types are regulated by protein kinase A and C [53], and three types are activated by Ca^2+^ or calmodulin [54]. Additionally, cytoskeleton disruption also enhanced some adenylyl cyclases [55]. A previous study revealed that adenylyl cyclase type 6, which is activated only by forskolin, was mechanoresponsive factor that increased inside the cells in response to shear stress [56]. However, it has not been reported that compression induced forskolin products or increased intracellular Ca^2+^. We did not observe collapse of actin fibers (Fig. 2) or an increase in intracellular Ca^2+^ after compressive loading to cells (data not shown). Further work is needed to investigate which adenylyl cyclases are activated in response to compressive loading.

Lastly, we note the specificity of the PKA inhibitor H-89. H-89 is currently used as a selective and potent inhibitor of protein kinase A (PKA). However, Davies et al. reported that H-89 inhibits at least eight kinases except for PKA [57]. It is also noted that ROCK is included in the kinases targeted by H-89. Thus, inhibition of other kinases excluding PKA (especially ROCK) may affect dephosphorylation of MRLC in response to compressive loading. Nevertheless, H-89 blocked phosphorylation of RhoA after cell compression. Within the targets of H-89, the only kinase that can regulate phosphorylation levels of RhoA is PKA. It was reported that phosphorylation of RhoA induces ROCK inactivation and affects the phosphorylation levels of MRLC [58]. Additionally, SQ22,536 also prevented RhoA phosphorylation and MRLC dephosphorylation in response to compressive loading. SQ22,536 inhibits some adenylyl cyclases, which results in inhibition of cAMP formation and PKA activity. Hence, we concluded that the adenylyl cyclase/cAMP/PKA signaling pathway is involved in compression-induced MRLC dephosphorylation.

## Supporting Information

S1 Fig Stretching cells did not affect phosphorylation of RhoA. (A) Influence of stretching C2C12 cells on phosphorylation of MRLC. MRLC phosphorylation 5 minutes after cell stretching was monitored by western blotting. GAPDH was used as a loading control. (B) Effects of stretch stress on RhoA activity. Active RhoA (RhoA-GTP) and total RhoA after 5 minutes of cell stretching were detected by western blotting. (C) Detection of phosphorylated RhoA under cell stretching. Phospho-RhoA and total RhoA were measured by western blotting.
(TIF)

S2 Fig Cell area homeostasis in response to compressive loading, is not dependent on adenylyl cyclase. Phase-contrast imaging was performed to examine the change in cell area after cell compression. Figures show a representative single cell before and after compressive strain for non-treatment (A) and under the treatment of adenylyl cyclase inhibitor (SQ22,536) (B). The yellow line shows the perimeter region of the cell. Numbers in upper left of the images are relative time (min) from the onset of compressive loading. Scale bar is 50 μm. (C) Quantitative analysis of cell area after cell compression. Cell area relative to the cell area before compressive loading was plotted. The black circles indicate the cell area under the non-treatment, and the red squares indicate the cell area under the treatment of SQ22,536. Quantification of cell area represents an average of 3 independent experiments including 12 individual cells, mean ± SEM.
(TIF)
