# Supplementary figures and images for: Compressive Stress Induces Dephosphorylation of the Myosin Regulatory Light Chain via RhoA Phosphorylation by the Adenylyl Cyclase/Protein Kinase A Signaling Pathway
Source: PLoS One. 2015 Mar 3;10(3):e0117937. doi: 10.1371/journal.pone.0117937 (PMC4348516; doi:10.1371/journal.pone.0117937)

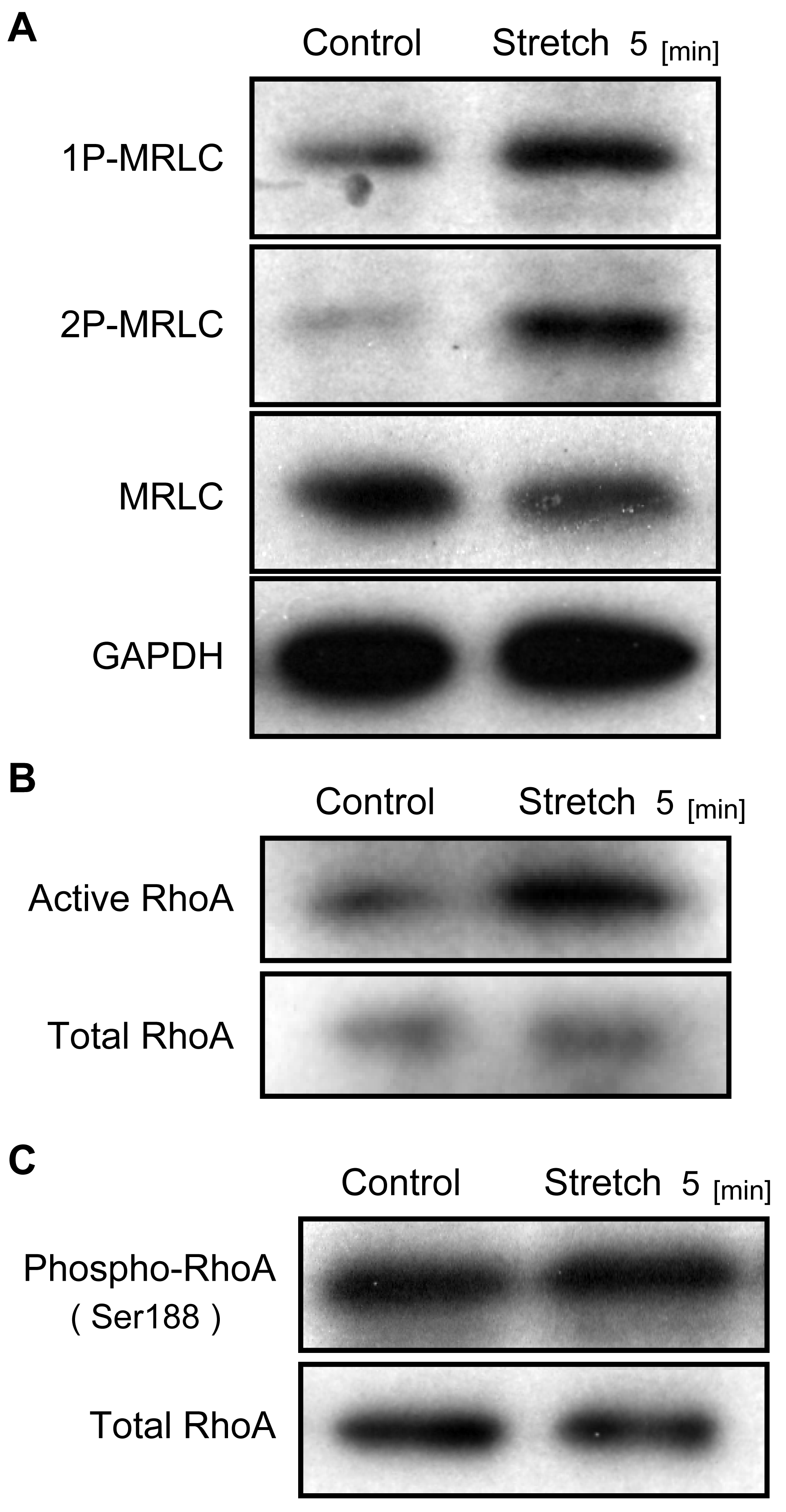

Supplement: S1 Fig — (A) Influence of stretching C2C12 cells on phosphorylation of MRLC. MRLC phosphorylation 5 minutes after cell stretching was monitored by western blotting. GAPDH was used as a loading control. (B) Effects of stretch stress on RhoA activity. Active RhoA (RhoA-GTP) and total RhoA after 5 minutes of cell stretching were detected by western blotting. (C) Detection of phosphorylated RhoA under cell stretching. Phospho-RhoA and total RhoA were measured by western blotting. (TIF) [file pone.0117937.s001.tif]

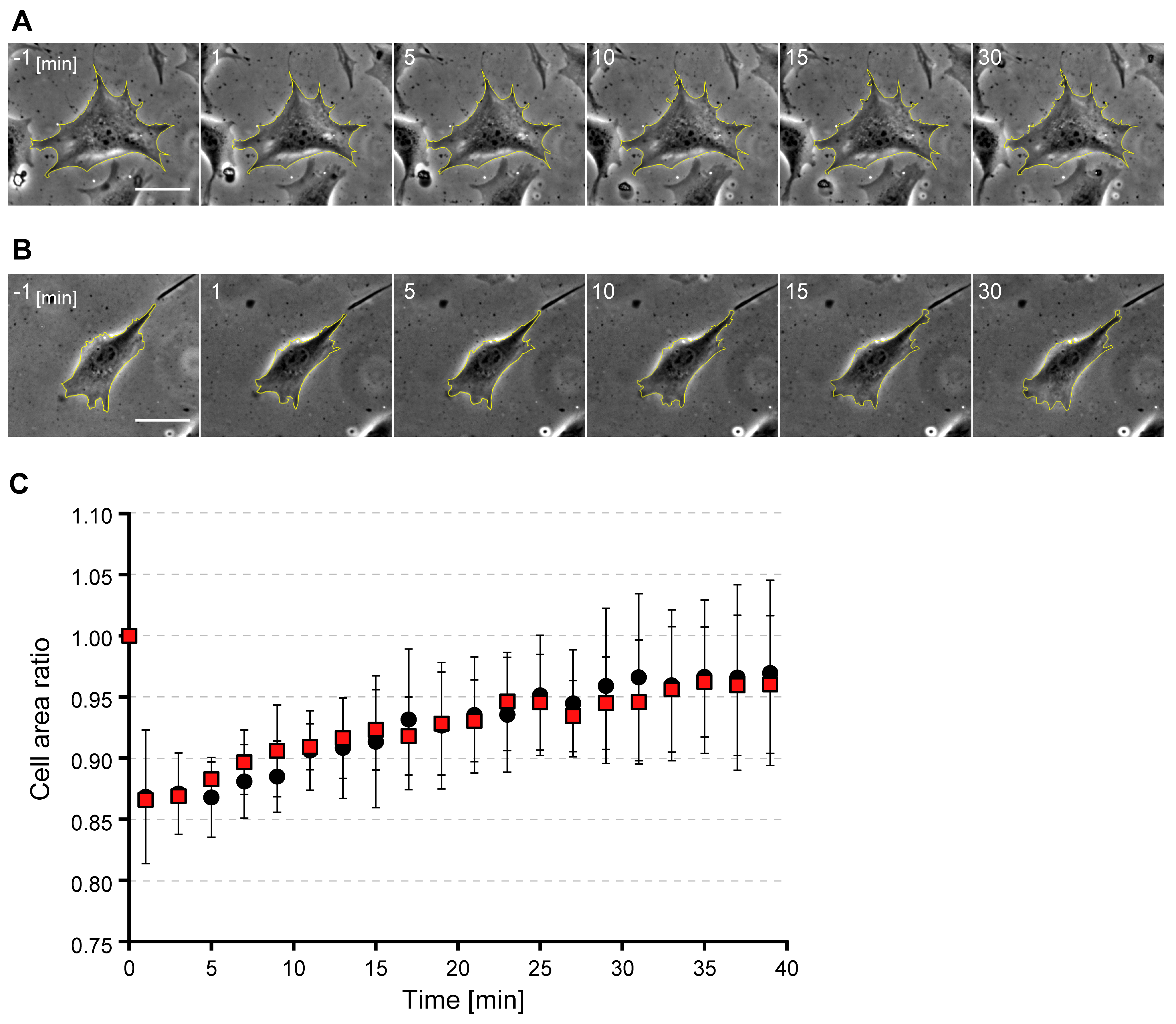

Supplement: S2 Fig — Phase-contrast imaging was performed to examine the change in cell area after cell compression. Figures show a representative single cell before and after compressive strain for non-treatment (A) and under the treatment of adenylyl cyclase inhibitor (SQ22,536) (B). The yellow line shows the perimeter region of the cell. Numbers in upper left of the images are relative time (min) from the onset of compressive loading. Scale bar is 50 μm. (C) Quantitative analysis of cell area after cell compression. Cell area relative to the cell area before compressive loading was plotted. The black circles indicate the cell area under the non-treatment, and the red squares indicate the cell area under the treatment of SQ22,536. Quantification of cell area represents an average of 3 independent experiments including 12 individual cells, mean ± SEM. (TIF) [file pone.0117937.s002.tif]
